# Genetic variability of the prion protein gene in Indonesian goat breeds

**DOI:** 10.1007/s11250-023-03486-7

**Published:** 2023-02-17

**Authors:** Suhendra Pakpahan, Rini Widayanti, Wayan Tunas Artama, I. Gede Suparta Budisatria, Gesine Lühken

**Affiliations:** 1 Research Center for Applied Zoology, Research Organization for Life Sciences, National Research and Innovation Agency (BRIN), Jl. Jakarta-Bogor Km.46, Cibinong, 16911 West Java Indonesia; 2 Department of Biochemistry and Molecular Biology, Faculty of Veterinary Medicine, Gadjah Mada University, Yogyakarta, 55281 Indonesia; 3 Department of Animal Production, Faculty of Animal Science, Gadjah Mada University, Yogyakarta, 55281 Indonesia; 4 Institute of Animal Breeding and Genetics, Justus-Liebig University, 35390 Giessen, Germany

**Keywords:** Scrapie, Prion protein, Indonesian goats, Genetic variability, Small ruminants

## Abstract

Scrapie is a naturally occurring transmissible spongiform encephalopathy in sheep and goats. Resistance or susceptibility of small ruminants to classical scrapie is influenced by polymorphisms in the prion protein gene (*PRNP*). *PRNP* variability in Indonesian indigenous goat breeds has not been investigated so far and therefore was the goal of this study. Sanger sequencing of the *PRNP* gene coding region in 72 goats of the seven Indonesian breeds Kacang, Gembrong, Samosir, Kejobong, Benggala, Jawarandu, and Peranakan Etawah revealed three amino acid substitutions, namely W102G, H143R, and S240P. Some silent mutations were also found at codons 42 (a/g), 138 (c/t), and 179 (g/t). The *PRNP* alleles K222 and D/S146 known to have significant protective effects on resistance to classical scrapie in goats were not detected. The allele R143, which may have a moderate protective effect, had a frequency of 12% among the analyzed Indonesian goat breeds. While R143 was missing in Kacang and Benggala, its frequency was highest in the breed Gembrong (32%). No scrapie cases have been reported in Indonesia until now. However, in the case that selection for protective *PRNP* variants would become a breeding goal, the analyzed breeds will not be very useful resources. Other goat breeds which are present in the country should be investigated regarding resistance to scrapie, too.

**Supplementary Information:**

The online version contains supplementary material available at 10.1007/s11250-023-03486-7.

## Introduction

Scrapie belongs to the transmissible spongiform encephalopathies (TSEs) like other similar diseases such as bovine spongiform encephalopathy (BSE) and human Creutzfeldt-Jakob-disease (CJD). TSEs are fatal degenerative disorders of the central nervous system, which are characterized by the accumulation of an abnormal isoform (PrP^sc^) of the cellular prion protein (PrP^c^) (Prusiner and DeArmond 1994). Scrapie has long been known in sheep and goats, with a less frequent occurrence in the latter (Chelle 1942; EFSA BIOHAZ Panel 2014). In 1998, a new, atypical type of scrapie was identified in sheep that differs in its epidemiological and biochemical characteristics from the long-known classical scrapie type (Benestad et al. 2003).

Compared to other species, the prion protein gene (*PRNP*) of sheep and goats is highly polymorphic, and *PRNP* variants, mostly amino acid substitutions, are associated with resistance or susceptibility to classical scrapie (overview given by Goldmann 2008). About 15 years ago, the European Union (EU), as well as other countries, started to select for sheep carrying the *PRNP* haplotype A_136_R_154_R_171_ (ARR), which was regarded to be resistant to classical scrapie, and to eliminate the other haplotypes, especially VRQ, which was suspected to be most susceptible (Dawson et al. 1998; Dubois et al. 2002; Baylis et al. 2004; Vitezica et al. 2005). In the EU, related breeding program requirements were integrated into several regulations. Unfortunately, the *PRNP* variants used in breeding do not protect sheep against atypical scrapie (Lühken et al. 2007).

In the past, scientific evidence regarding the association between PRNP variants in goats and resistance or susceptibility to scrapie has been insufficient to pursue a strategy similar to that used in sheep, particularly due to the lower number of scrapie cases in goats compared to sheep (Vaccari et al. 2009). However, in the last decade, further scientific evidence was obtained on this subject, which was evaluated by the European Food Safety Authority (EFSA). In this report, it was concluded that the results of various studies indicate that nine amino acid substitutions (codons 127, 142, 143, 145, 146, 154, 211, and 222) provide some degree of resistance to classical scrapie. Both the quality and reliability of the available field and experimental data were considered robust enough to conclude that the K222, D146, and S146 alleles confer significant genetic resistance to classical scrapie strains naturally occurring in the EU goat population. The authors of this large meta-analysis recommend to consider breeding for resistance as an effective tool for controlling classical scrapie in goats (EFSA BIOHAZ Panel 2017). A strong protective effect of S146 and K222 was already confirmed for goats heterozygous with one of these alleles, surviving oral inoculation with classical scrapie for more than 6 years, while non-carriers of these alleles showed clinical symptoms after an average of 2 years after inoculation (Cinar et al. 2018).

Unfortunately, the protective PRNP allele K222 was found only at low frequencies in the cosmopolitan goat breeds Alpine and Saanen in various countries like France, Italy, Spain, the Netherlands, the UK, and the USA. In contrast, higher frequencies of the protective allele D146 were observed in populations of the also worldwide distributed Boer goat in the Netherlands, the UK, and the USA. For the Toggenburg breed, a higher frequency of K222 was determined in the Netherlands, but not in the UK and the USA, which is maybe due to founder effects (Acutis et al. 2008; White et al. 2008; Barillet et al. 2009; Goldmann et al. 2011, 2016; Acín et al. 2013; Windig et al. 2016).

Higher frequencies of K222 or S/D146 were found in single local breeds, as, e.g., in the Garganica breed of southern Italy (Acutis et al. 2008), in the Small East African breed from Tanzania (Kipanyula et al. 2014), and in Damascus related breeds from Cyprus and Turkey (Meydan et al. 2017; EFSA 2012). These findings aroused interest in studying the *PRNP* variability of other local goat breeds from other regions of the world.

For Asian and Eurasian goat populations, e.g., from China, Japan, Korea, Turkey, and Pakistan, data on frequencies of *PRNP* polymorphisms are available (Kurosaki et al. 2005; Babar et al. 2008; Hussain et al. 2011; Zhou et al. 2013; Hassan et al. 2016; Kim et al. 2019; Meydan et al. 2017). In contrast, even since Indonesia represents a large southern part of the Asian continent, no data on *PRNP* variability of the Indonesian goat population has been published until now. Therefore, the aim of this study was to analyze for the first time the *PRNP* coding region of seven Indonesian goat breeds in order to assess their susceptibility status toward classical scrapie as well as their potential to be a resource for protective *PRNP* alleles.

## Materials and methods

### Collection of animal samples

**Fig. 1:**
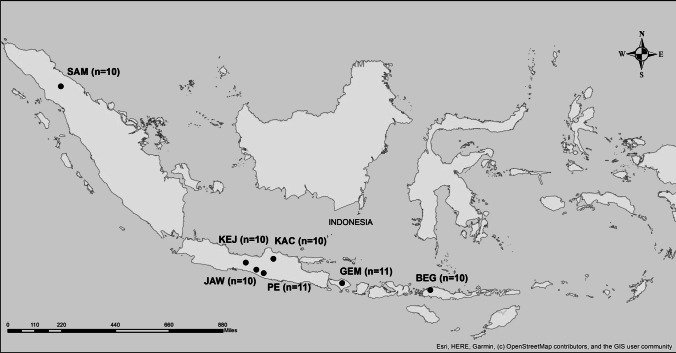
Locations of sampling Indonesian goat breeds. The map was created using ArcMap version 10.5. The OpenStreetMap provides free geographic data and is available under the Open Database Licence (https://www.openstreetmap.org/copyright)

A total of 72 samples (10–11 samples per breed) from goats belonging to seven breeds were collected from different farms in several provinces in Indonesia (Fig. 1). The samples were collected in the regions in which these breeds usually are bred.

These Indonesian goat breeds included the three native breeds Kacang (Central Java), Gembrong (Bali Island), and Samosir (North Sumatera), as well as the cross breeds Kejobong (Central Java), Benggala (Nusa Tenggara Timur), Jawarandu, and Peranakan Etawah (both Yogyakarta). In the individual farms with herd sizes of about 20–30 animals, 3–5 unrelated animals were sampled each. Two-thirds of the sampled goats were female. Depending on the management system, animals were kept in groups on pasture or indoors separated from each other. Only apparently healthy animals with a good body condition were sampled. Blood samples were collected from the jugular vein using 3 ml EDTA monovettes. All individuals were identified based on morphological characteristics and were considered to be genetically unrelated.

### DNA extraction, PCR amplification, and sequencing of *PRNP*

Genomic DNA was isolated using a commercial extraction kit (Blood DNA isolation kit, Geneaid Biotech Ltd. New Taipei City, Taiwan) as recommended by the manufacturer. Quantity and quality of extracted DNA were checked using a NanoDrop 2000 spectrophotometer (VWR, Darmstadt, Germany). Polymerase chain reaction (PCR) for the amplification of a 970 bp fragment containing the complete *PRNP* coding region was done as described in Lühken et al. (2007) for ovine *PRNP*. Due to differences between sheep and goat *PRNP* sequences in the binding region of the forward primer, it was necessary to modify the forward primer (TGCCASTGCTATGCAGTCATT, changed nucleotides underlined). PCR program: An initial denaturing stage at 95 °C for 5 min was followed by 35 cycles at 94 °C for 30 s, 55 °C for 90 s, and 72 °C for 60 s, with a final step at 72 °C for 10 min. The PCR products were purified using a commercial kit (MSB Spin PCRapace, Stratec Molecular, Berlin, Germany), diluted to 10 ng/µl after quantification by agarose gel electrophoresis and sent to LGC Genomics GmbH (Berlin, Germany) for Sanger sequencing using the reverse PCR primer. The resulting chromatograms were analyzed with Chromas 1.74 (Technelysium Pty Ltd, South Brisbane, Australia). The GenBank sequence EF192309.2 was used as a reference sequence of the goat *PRNP* gene, representing the wild type (Acutis et al. 2008).

### Calculation of genotype and allele frequencies: chi-square/Fisher’s exact tests

The genotype and allele frequencies of the identified *PRNP* variants were calculated based on the numbers of animals with a certain genotype of a variable *PRNP* position divided by the total number of animals, or the numbers of chromosomes with a certain allele of a variable *PRNP* position divided by the total number of chromosomes, respectively. Calculations were done over all samples as well as within single breeds. Chi-square or, in the case of numbers below five, Fisher’s exact test was used to test for significant (*p* ≤ 0.05) differences in the distribution of alleles between pairs of breeds.

## Results

Six polymorphisms were observed in the coding *PRNP* region of the analyzed indigenous Indonesian goat breeds, namely the three amino acid substitutions W102G, H143R, and P240S, as well as the three synonymous mutations P42P, S138S, and V179V. All seven breeds were polymorphic for codons 42, 138, and 240, whereas codons 143, 179, and 102 were only variable in five (GEM, JAW, KEJ, PE, SAM), three (JAW, KEJ, PE), and a single breed (BEG), respectively. The W102G polymorphism occurred only in the BEG breed. Detailed genotype and allele frequencies of all identified variants over all breeds and within single breeds are shown in Tables 1 and 2. For all polymorphic positions, alleles were significantly different distributed at least between a single pair of breeds (P240S) up to seven pairs of breeds (H143R and V197V). All results of chi-square or Fisher’s exact tests are shown in Table S2.

**Table 1:** Genotype frequencies (numbers) of variable positions in the *PRNP* coding region in Indonesian goat breeds. Genotypes: Uppercase letters designate amino acids, lowercase letters designate nucleotides.

Genotypes at *PRNP *codons	Indonesian goat breeds							
	All	BEG	GEM	JAW	KAC	KEJ	PEE	SAM
P42P								
aa	0.46 (33)	0.50 (5)	0.36 (4)	0.30 (3)	0.50 (5)	0.50 (5)	0.36 (4)	0.70 (7)
ag	0.32 (23)	0.30 (3)	0.55 (6)	0.40 (4)	0.30 (3)	0.20 (2)	0.18 (2)	0.30 (3)
gg	0.22 (16)	0.20 (2)	0.09 (1)	0.30 (3)	0.20 (2)	0.30 (3)	0.46 (5)	0.00 (0)
W102G								
WW	0.94 (68)	0.60 (6)	1.00 (11)	1.00 (10)	1.00 (10)	1.00 (10)	1.00 (11)	1.00 (10)
GW	0.03 (2)	0.20 (2)	(0.00) (0)	(0.00) (0)	(0.00) (0)	(0.00) (0)	(0.00) (0)	(0.00) (0)
GG	0.03 (2)	0.20 (2)	(0.00) (0)	(0.00) (0)	(0.00) (0)	(0.00) (0)	(0.00) (0)	(0.00) (0)
S138S								
cc	0.42 (30)	0.50 (5)	0.36 (4)	0.30 (3)	0.50 (5)	0.50 (5)	0.18 (2)	0.60 (6)
ct	0.36 (26)	0.30 (3)	0.55 (6)	0.40 (4)	0.30 (3)	0.20 (2)	0.36 (4)	0.40 (4)
tt	0.22 (16)	0.20 (2)	0.09 (1)	0.30 (3)	0.20 (2)	0.30 (3)	0.46 (5)	(0.00) (0)
H143R								
HH	0.79 (57)	1.00 (10)	0.36 (4)	0.60 (6)	1.00 (10)	0.80 (8)	0.91 (10)	0.90 (9)
HR	0.17 (12)	(0.00) (0)	0.64 (7)	0.20 (2)	(0.00) (0)	0.10 (1)	0.09 (1)	0.10 (1)
RR	0.04 (3)	(0.00) (0)	(0.00) (0)	0.20 (2)	(0.00) (0)	0.10 (1)	(0.00) (0)	(0.00) (0)
V179V								
gg	0.88 (63)	1.00 (10)	1.00 (11)	0.80 (8)	1.00 (10)	0.70 (7)	0.64 (7)	1.00 (10)
gt	0.04 (3)	(0.00) (0)	(0.00) (0)	0.10 (1)	(0.00) (0)	0.10 (1)	0.09 (1)	(0.00) (0)
tt	0.08 (6)	(0.00) (0)	(0.00) (0)	0.10 (1)	(0.00) (0)	0.20 (2)	0.27 (3)	(0.00) (0)
P240S								
PP	0.22 (16)	0.20 (2)	0.09 (1)	0.30 (3)	0.20 (2)	0.30 (3)	0.46 (5)	(0.00) (0)
PS	0.36 (26)	0.30 (3)	0.55 (6)	0.40 (4)	0.20 (2)	0.10 (1)	0.36 (4)	0.60 (6)
SS	0.42 (30)	0.50 (5)	0.36 (4)	0.30 (3)	0.60 (6)	0.60 (6)	0.18 (2)	0.40 (4)

*BEG*, Bengala; *GEM*, Gembrong; *JAW*, Jawarandu; *KAC*, Kacang; *KEJ*, Kejobong; *PEE*, Peranakan Etawah; *SAM*, Samosir

**Table 2:** Allele frequencies of variable positions in the *PRNP* coding region in Indonesian goat breeds (allele numbers are shown in Table S1). Alleles: Uppercase letters designate amino acids, lowercase letters designate nucleotides.

Alleles at *PRNP* codons	Indonesian goat breeds							
	All	BEG	GEM	JAW	KAC	KEJ	PEE	SAM
P42P								
a	0.62	0.65	0.64	0.50	0.65	0.60	0.44	0.85
g	0.38	0.35	0.36	0.50	0.35	0.40	0.56	0.15
W102G								
W	0.96	0.70	1.00	1.00	1.00	1.00	1.00	1.00
G	0.04	0.30	0.00	0.00	0.00	0.00	0.00	0.00
S138S								
c	0.60	0.65	0.64	0.50	0.65	0.60	0.364	0.80
t	0.40	0.35	036	0.50	0.35	0.40	0.636	0.20
H143R								
H	0.88	1.00	0.68	0.70	1.00	0.85	0.96	0.95
R	0.12	0.00	0.32	0.30	0.00	0.15	0.04	0.05
V179V								
g	0.90	1.00	1.00	0.85	1.00	0.75	0.68	1.00
t	0.10	0.00	0.00	0.15	0.00	0.25	0.32	0.00
P240S								
P	0.40	0.35	0.36	0.50	0.30	0.35	0.64	0.30
S	0.60	0.65	0.64	0.50	0.70	0.65	0.36	0.70

*BEG*, Bengala; *GEM*, Gembrong; *JAW*, Jawarandu; *KAC*, Kacang; *KEJ*, Kejobong; *PEE*, Peranakan Etawah; *SAM*, Samosir

## Discussion

As in many other countries all over the world, the occurrence of scrapie is not monitored in Indonesia. The Indonesian goat population includes many native, crossbred, and foreign breeds (Pakpahan et al. 2016). To our knowledge, this is the first study on variability of the coding region of the *PRNP* gene in Indonesian goat breeds. By sequencing the target region in 72 goats of seven different breeds, in total six polymorphic positions were detected, three of them causing amino acid substitutions. In all analyzed breeds, at least three *PRNP* codons were variable, always including the amino acid substitution at codon 240.

Among the *PRNP* variants detected in Indonesian goats, only R143 belongs to the nine *PRNP* variants which are supposed to confer some degree of resistance to classical scrapie. In the analyzed Indonesian goat breeds, the R143 allele was identified at a frequency of 12% among all breeds, ranging from 0% in two breeds up to 32% in GEM. Epidemiological data from Italy, France, Greece, and Cyprus indicated an association of the 143R allele with lower susceptibility to classical scrapie (Billinis et al. 2002; Acutis et al. 2006; Vaccari et al. 2006; Papasavva-Stylianou et al. 2011; Fragkiadaki et al. 2011). For example, Billinis et al. (2002) observed a significantly higher proportion of scrapie-positive goats among those carrying the genotype HH at position 143 compared to goats with genotypes HR or RR (*P* = 0.012). However, 143R did not show a protective effect in vitro conversion experiments (Eiden et al. 2011). Until now, results from experimental inoculation with classical scrapie have only been reported for a single 143HR goat (Goldmann et al. 1996). According to this, R143 confers a slightly longer incubation period compared to H143, but a significantly shorter incubation period than goats with M142 (together with H143). Considering all available data, goats with R143 may be better protected compared to wild-type animals, but the potential protective effect against classical scrapie seems to be much lower compared to other *PRNP* alleles, especially K222 and D/S146 (EFSA BIOHAZ Panel 2017).

As a further amino acid substitution in *PRNP*, W102G was identified in only one of the Indonesian breeds (BEG). To our knowledge, G102 is a very rare allele, and therefore there is not enough epidemiological field data available to study a potential protective effect of G102 against scrapie in goats. An experimentally scrapie-infected goat heterozygous for three octapeptide repeats in combination with G102 (while the other chromosome carried the wild type, which is five octapeptide repeats, in combination with W102) succumbed to scrapie only after a significantly prolonged incubation period compared with goats homozygous for the wild-type allele combination (Goldmann et al. 1998). However, it is not clear if this could be attributed to the missing of two octapeptide repeat sequences, to the amino acid substitution at position 102, or a combined effect of both mutations. The loss of two octapeptides, as well as the amino acid substitution at codon 102 probably, modulates the copper ion binding function of the prion protein (Stöckel et al. 1998). Therefore, a theoretical effect on scrapie susceptibility of both of the two types of mutations could be related to the same biochemical mechanism. Finally, P240S was observed in all analyzed breeds as the third and last amino acid substitution in the prion protein of Indonesian goats. In all investigated goat populations so far, P and S at position 240 seem to be more or less equally distributed. This is also reflected in the Indonesian goat breeds, with a slightly higher frequency of P in some breeds and of S in others. No association between the length of disease incubation and the different codon 240 genotypes was found in scrapie inoculation experiments (Goldmann et al. 1996). Moreover, it is likely that, as has been demonstrated for rodent PrP (Stahl et al. 1990), the C-terminal region of goat PrP, including amino acid 240, is removed during the post-translational attachment of a glycoinositol phospholipid tail.

Taken together, in the analyzed Indonesian goat breeds, three amino acid substitutions have been identified (W102G, H143R, S240P). Only the R143 allele may have a moderate effect on resistance against classical scrapie in goats (EFSA BIOHAZ Panel 2017). The R143 allele is also present in some goat breeds in other Asian/Eurasian countries, e.g., at a frequency of about 15% in the Pakistani breeds Kamori and Local Hairy, and 29% and 37% in southern and northern Chinese goat populations, respectively (Zhou et al. 2013). The *PRNP* alleles with known significantly protective effects, K222 or D/S 146, which were not detected in the samples of the analyzed Indonesian breeds, were found in other Asian or Eurasian populations at very low frequencies, e.g., in some Chinese, Japanese, Korean, Pakistani, and Turkish breeds. Only in the Turkish Halep breed, the S146 frequency was higher with 28% (Hussain et al. 2011; Zhou et al. 2013; Hassan et al. 2016; Meydan et al. 2017; Kim et al. 2019; Akis et al. 2020).

Due to insufficient passive surveillance systems, the actual prevalence of scrapie remains unknown in many countries (Curcio et al. 2016); this is also the case in Indonesia, where no scrapie cases have been reported until now. Therefore, their apparent genetic susceptibility based on *PRNP* polymorphisms seems not to pose an acute risk to the goat breeds studied. However, based on current knowledge, it has to be concluded that these breeds are no relevant resource for resistance breeding programs in the case this might be necessary in the future. However, in order to represent each region of the Indonesian archipelago, research on Lakor, Marica, Bligon, Muara, and Kosta goat breeds, other Indonesian goat breeds which were not included in this study, should be done, using additional samples and other sampling locations.

## Supplementary Information

Below is the link to the electronic supplementary material.

Supplementary file1 (PDF 334 KB)

## Data Availability

The datasets generated during the current study are available from the corresponding author on reasonable request.
