# Supplementary material for: Genetic variability of the prion protein gene in Indonesian goat breeds
Source: Trop Anim Health Prod. 2023 Feb 17;55(2):87. doi: 10.1007/s11250-023-03486-7 (PMC9938069; doi:10.1007/s11250-023-03486-7)
Supplement: Supplementary file 1 — Supplementary file1 (PDF 334 KB) [file 11250_2023_3486_MOESM1_ESM.pdf]

Table S1 Numbers of *PRNP* alleles in Indonesian goat breeds

Alleles at *PRNP* codons	Indonesian goat breeds							
	All	BEG	GEM	JAW	KAC	KEJ	PEE	SAM
P42P								
a	89	13	14	10	13	12	10	17
g	55	7	8	10	7	8	12	3
W102G								
W	138	14	22	20	20	20	22	20
G	6	6	0	0	0	0	0	0
S138S								
c	86	13	14	10	13	12	8	16
t	58	7	8	10	7	8	14	4
H143R								
H	126	20	15	14	20	17	21	19
R	18	0	7	6	0	3	1	1
V179V								
g	129	20	22	17	20	15	15	20
t	15	0	0	3	0	5	7	0
P240S								
P	58	7	8	10	6	7	14	6
S	86	13	14	10	14	13	8	14

BEG = Bengala; GEM = Gembrong; JAW=Jawarandu; KAC=Kacang; KEJ=Kejobong; PEE= Peranakan Etawah; SAM=Samosir

Table S2 Results (p values) of chi square or Fisher's exact test for difference of PRNP allele distributions between pairs of breeds (p values ≤ 0.05 are shown in bold)

**P42P**	BEG	GEM	JAW	KAC	KEJ	PEE
BEG						
GEM	1.000					
JAW	0.337	0.372				
KAC	1.000	1.000	0.337			
KEJ	0.744	1.000	0.525	0.744		
PEE	0.204	0.226	0.768	0.204	0.346	
SAM	0.104	0.085	**0.018**	0.104	0.062	**0.007**

**W102G**	BEG	GEM	JAW	KAC	KEJ	PEE
BEG						
GEM	**0.007**					
JAW	**0.010**	1.000				
KAC	**0.010**	1.000	1.000			
KEJ	**0.010**	1.000	1.000	1.000		
PEE	**0.007**	1.000	1.000	1.000	1.000	
SAM	**0.010**	1.000	1.000	1.000	1.000	1.000

**S138S**	BEG	GEM	JAW	KAC	KEJ	PEE
BEG						
GEM	1.000					
JAW	0.337	0.372				
KAC	1.000	1.000	0.337			
KEJ	0.744	1.000	0.525	0.744		
PEE	0.064	0.070	0.372	0.064	0.126	
SAM	0.162	0.241	**0.039**	0.162	0.109	**0.004**

**H143R**	BEG	GEM	JAW	KAC	KEJ	PEE
BEG						
GEM	**0.001**					
JAW	**0.002**	1.000				
KAC	1.000	**0.001**	**0.010**			
KEJ	0.115	0.132	0.162	0.115		
PEE	0.524	**0.021**	0.312	0.524	0.065	
SAM	0.500	**0.029**	**0.042**	0.500	0.081	0.511

BEG = Bengala; GEM = Gembrong; JAW=Jawarandu; KAC=Kacang; KEJ=Kejobong; PEE= Peranakan Etawah; SAM=Samosir

Table S2 (continued)

**V197V**	BEG	GEM	JAW	KAC	KEJ	PEE
BEG						
GEM	1.000					
JAW	0.115	0.099				
KAC	1.000	1.000	0.115			
KEJ	**0.024**	0.018	0.230	**0.024**		
PEE	**0.006**	**0.004**	0.132	**0.006**	0.625	
SAM	1.000	1.000	0.115	1.000	**0.024**	**0.006**

**P240S**	BEG	GEM	JAW	KAC	KEJ	PEE
BEG						
GEM	1.000					
JAW	0.337	0.372				
KAC	0.736	0.819	0.197			
KEJ	1.000	1.000	0.337	0.736		
PEE	0.064	0.070	0.372	1.000	0.064	
SAM	0.736	0.662	0.197	1.000	0.736	**0.029**

BEG = Bengala; GEM = Gembrong; JAW=Jawarandu; KAC=Kacang; KEJ=Kejobong; PEE= Peranakan Etawah; SAM=Samosir
